# Transgenic *Nicotiana tabacum* seeds expressing the *Mycobacterium tuberculosis* Alanine- and Proline-rich antigen

**DOI:** 10.1186/s13568-018-0708-y

**Published:** 2018-10-31

**Authors:** Diego G. Módolo, Cynthia S. Horn, José S. M. Soares, José A. Yunes, Leila M. Lima, Sylvia M. de Sousa, Marcelo Menossi

**Affiliations:** 1 Laboratório de Genoma Funcional, Departamento de Genética, Evolução, Microbiologia e Imunologia, Instituto de Biologia, Universidade Estadual de Campinas, Rua Monteiro Lobato, 255, Barão Geraldo, PO. Box: 6109, Campinas, SP 13083-970 Brazil; 2 Laboratório de Genética, Instituto Butantan, São Paulo, SP 05503-900 Brazil; 3 Laboratório de Imunologia e Imunogenética de Doenças Infecciosas, Instituto Nacional de Infectologia Evandro Chagas, Fundação Oswaldo Cruz, Rio de Janeiro, RJ 21040-900 Brazil; 4 Laboratório de Biologia Molecular, Centro Infantil de Investigações Hematológicas Dr Domingos A Boldrini, Campinas, SP 13083-210 Brazil; 5 Departamento de Bioquímica e Biologia Molecular, Instituto Oswaldo Cruz, Fundação Oswaldo Cruz, Rio de Janeiro, RJ 21040-900 Brazil; 6 Centro Nacional de Pesquisa de Milho e Sorgo, Empresa Brasileira de Pesquisa Agropecuária, Sete Lagoas, MG 35701-970 Brazil

**Keywords:** *Mycobacterium tuberculosis*, APA, 45/47 kDa, Tobacco, Alanine- and Proline-rich antigen, Seed

## Abstract

The glycoprotein APA (Alanine- and Proline-rich Antigen, a 45/47 kDa antigen complex, Rv1860) is considered as a major immunodominant antigen secreted by *M.* *tuberculosis*. This antigen has proved to be highly immunogenic in experimental models and humans, presenting a significant potential for further development of a new vaccine for tuberculosis. Glycosylation plays a key role in the immunogenicity of the APA protein. Because plants are known to promote post-translational modification such as glycosylation and to be one of the most economic and safe hosts for recombinant protein expression, we have over expressed the APA protein in transgenic tobacco plants aiming to produce a glycosylated version of the protein. Seeds are known to be a well-suited organ to accumulate recombinant proteins, due to low protease activity and higher protein stability. We used a seed-specific promoter from sorghum, a signal peptide to target the protein to the endoplasmic reticulum and ultimately in the protein storage vacuoles. We show that the recombinant protein accumulated in the seeds had similar isoelectric point and molecular weight compared with the native protein. These findings demonstrate the ability of tobacco plants to produce glycosylated APA protein, opening the way for the development of secure, effective and versatile vaccines or therapeutic proteins against tuberculosis.

## Introduction

Tuberculosis (TB), caused by *Mycobacterium tuberculosis*, is the first cause of death due to a single infectious agent in the world, leading to approximately 1.8 million deaths annually, and it was estimated that in 2015 there were 10.4 million TB cases globally (WHO 2017). HIV co-infection, which dramatically compromises host resistance, and the emergence of multidrug-resistance *M. tuberculosis* strains strongly collaborate for the increase in TB incidence (Maartens and Wilkinson 2007).

The only available vaccine against TB is the live attenuated *M. bovis* strain Bacillus Calmette–Guérin (BCG), which provides effective protection against severe forms of childhood TB (meningeal and miliary TB). However, it fails to protect against the most frequent occurrence of the disease, pulmonary TB, in adults, and has not reduced the global burden of TB (Colditz et al. 1994; Fine 2001). Moreover, the use of a live vaccine (BCG) has the risk of developing pathologic effects in immunocompromised individuals (Norouzi et al. 2012). Therefore, better TB vaccines are urgently needed.

Antigenic proteins actively secreted during *M. tuberculosis* growth are the best suited targets to develop a new subunit vaccine against TB (Andersen 1994; Garapin et al. 2001; Kumar et al. 2003; Ferraz et al. 2004; Sable et al. 2011; Carlétti et al. 2013). Alanine- and Proline-rich Antigen (APA), also known as a 45/47 kDa antigen complex, is a highly immunogenic glycoprotein secreted by the *M. tuberculosis* complex (Laqueyrerie et al. 1995; Berrêdo-Pinho et al. 2011). Despite the APA migration in SDS-PAGE gel shows bands of 45/47 kDa, this antigen when analyzed by mass spectrometry has a molecular weight of approximately 28 kDa, with the high percentage of proline and the presence of carbohydrates found in APA causing an abnormal migration on SDS-PAGE (Dobos et al. 1996; Horn et al. 1999). The 45 kDa band observed in SDS-PAGE can be due to the presence of truncated APA protein molecules, with a C-terminal modification, that are co-purified with the 47 kDa protein (Romain et al. 1999), and other study describes that the presence of the 45 kDa band may be due to a differentiation in the glycosylation pattern of this protein (Lara et al. 2004).

The post-translational modification of *M. tuberculosis* APA antigen includes a complex O-mannosylation of Thr residues in N- and C-terminal domains of the protein (Dobos et al. 1996), specifically, multiple α (1,2) mannose residues such as mannose, mannobiose, mannotriose were identified in Thr49, Thr57, Thr66 and Thr316 (Dobos et al. 1995; 1996). Moreover, an additional glycosylation of one, two or three hexoses between Thr313, Thr315, Thr316 and Thr318 has been observed (Smith et al. 2014). Mass spectrometry analysis show a variable pattern of mannosylation of APA molecules, and while a small number of native APA are not glycosylated, some have one to nine mannose residues, with a majority of glycoform bearing 6–8 mannose residues (Horn et al. 1999).

The absence or low levels of glycosylation, as demonstrated in the recombinant expression of APA in *E. coli* and *M. smegmatis,* respectively, leads to a great reduction in its immunological properties when compared with the native (glycosylated) protein, suggesting that glycosylation is essential to keep the immune activity of the APA antigen (Horn et al. 1999).

APA has been described as an adhesion molecule that interacts with the surfactant protein A in the lung, and it has been observed that this interaction is dependent on the glycosylation of APA (Ragas et al. 2007). Similarly, APA is considered a fibronectin attachment protein (FAP) which binds to bladder tumour cells, having an important role in the treatment of bladder cancer, being quite effective as an alternative therapy to BCG for the treatment of this disease (Sinn et al. 2008). Furthermore, monoclonal antibody against APA has been previously shown to abrogate the attachment and internalization of BCG by human bladder tumour cells, and the stable binding of BCG to bladder mucosa via FAP was necessary for the expression of BCG-induced anti-tumour activity (Kuroda et al. 1993; Zhao et al. 2000). Thus, the glycosylation and the fibronectin-binding domain could reinforce its interaction with the host.

Transgenic plants present enormous potential as a cost-effective and safe platform for large scale production of vaccines and therapeutic proteins (Hefferon 2012). Various antigens have been successfully expressed in plants and have been shown to retain their native functionalities (Scissum Gunn et al. 2012). The production of vaccines against TB in plants has increased over the years, and recent studies have demonstrated the efficient expression of the early secretory antigenic target (ESAT-6) in plant cells (Zelada et al. 2006; Dorokhov et al. 2007; Zeng et al. 2008; Lakshmi et al. 2013), indicating plants are able to produce *M. tuberculosis* antigens.

The ability of plant cells to promote post-translational processing makes this model even more interesting than those based in prokaryotes (Bednarek and Raikhel 1992). The glycosylation of proteins has several functions, such as the maintenance of the three-dimensional structure and increased stability (Benz and Schmidt 2002; Lee et al. 2015). Plants have a wide range of enzymes involved in glycosylation (Strasser 2016). The pattern of protein glycosylation performed by plant cells differs slightly from that observed in human cells. The first stage of protein glycosylation is similar in all eukaryotic organisms, with the binding of an oligosaccharide precursor Man_3_GlcNAc_2_ in the endoplasmic reticulum (ER). The nascent N-glycan is then processed by enzymes contained in the Golgi apparatus and these enzymes differ between plants, mammals, insects and yeasts, leading to a differentiation of the oligosaccharide structure (Peters and Stoger 2011). In plants, *N*-glycosylation comprises the addition of an α (1,3) fucose and a β (1,2) xylose in the core structure, while in humans an α (1,6) fucose, β (1,4) galactose and *N*-acetylneuraminic acid is added to the glycoconjugate structure. In insect cells, the glycoconjugate has a smaller core of N-glycans, with low sialylation, while yeasts have a completely different pattern, with only mannose-containing glycan (Strasser 2016).

Regarding O-glycosylation, plants transfer only one GlcNAc to Ser/Thr residues (O-GlcNAcylation), in the same way as in mammals. In addition, plants may add a galactose (Gal) to serine residues and arabinose chains (Ara) to hydroxyproline residues (Strasser 2016). Although plant-specific N-glycans, when injected subcutaneously, do not cause any adverse effects and no alteration of the immune response in humans (McCormick et al. 2008), alternatives are being developed to humanize the glycosylation pattern in plants when necessary, as the knockout of genes encoding enzymes responsible for the addition of specific plants glycans (Peters and Stoger 2011). Tobacco is a model system for recombinant protein production and is the most used species for the production of recombinant proteins at the laboratory scale (Cramer et al. 1999; Fischer and Emans 2000). The main advantages of tobacco include: advanced technology of gene transfer and expression, production of a high amount of seeds, which in turn also allows a fast scale-up of the number of transgenic plants in just a few generations (when the gene integration is stable), the availability of infrastructure for large-scale processing, and last, but not least, tobacco it is not used as food (Fischer and Emans 2000).

Seeds are the organ of choice to direct the expression of recombinant proteins when it is desirable to accumulate large amounts of proteins. They are designed for the synthesis and storage of proteins, giving an advantage over green tissues and tubers, in which the protein content is lower (De Jaeger et al. 2002; Shukla and Thömmes 2010). Moreover, seeds have low levels of non-enzymatic hydrolysis and protein degradation (Fischer et al. 2009). Therefore, recombinant proteins accumulated in the seeds remain stable and functional for several years, even after storage at room temperature (Stöger et al. 2000; Foley et al. 2011).

It is of great interest to develop systems that produce recombinant proteins with biological properties similar to native proteins (Woodard et al. 2004). Considering the relevance of glycosylation patterns for some proteins and also the seek of an efficient and safe production of recombinant proteins without human pathogens in a cost effective way, the use of transgenic plants is a good alternative (Ma et al. 2003).

Given the existence of a glycosylation system in plants, there is a possibility that the APA protein produced in transgenic plants, such as tobacco, would have a glycosylation profile, probably different from mycobacteria, but that could present in vivo immunogenic activity. Therefore, the focus of this study was to produce transgenic tobacco plants that express the recombinant protein APA in their seeds. For this purpose, we used an expression vector with a seed-specific promoter for γ-kafirin from sorghum, followed by a signal peptide of α-coixin to direct the recombinant protein to the endoplasmic reticulum (for protein glycosylation in the secretory pathway) and consequent accumulation of APA in protein storage vacuoles. We obtained the partially purified recombinant protein and we present evidence of recombinant protein glycosylation due to its affinity for binding to Concanavalin A and due to the presence of multiple isoforms in two-dimensional electrophoresis. This open new venues for the production of a recombinant vaccine for tuberculosis.

## Materials and methods

### Plant materials and culture conditions

*Nicotiana tabacum* SR1 (Maliga et al. 1973) seeds were surface-sterilized in 70% ethanol for 1 min and rinsed three times with sterile distilled water. Then they were incubated in 1% NaOH for 20 min and rinsed 5 times with sterile distilled water. Sterilized seeds were then germinated on MS medium (Murashige and Skoog 1962) containing sucrose 30 g/L, MS vitamin 1 mL/L and Phytagel 2.5 g/L in Petri dishes. Seedlings were transferred to glass pots with the same culture medium for 3 weeks at 25 °C with a photoperiod of 16 h day/light.

### Construction of plant expression vector

**Fig. 1:**
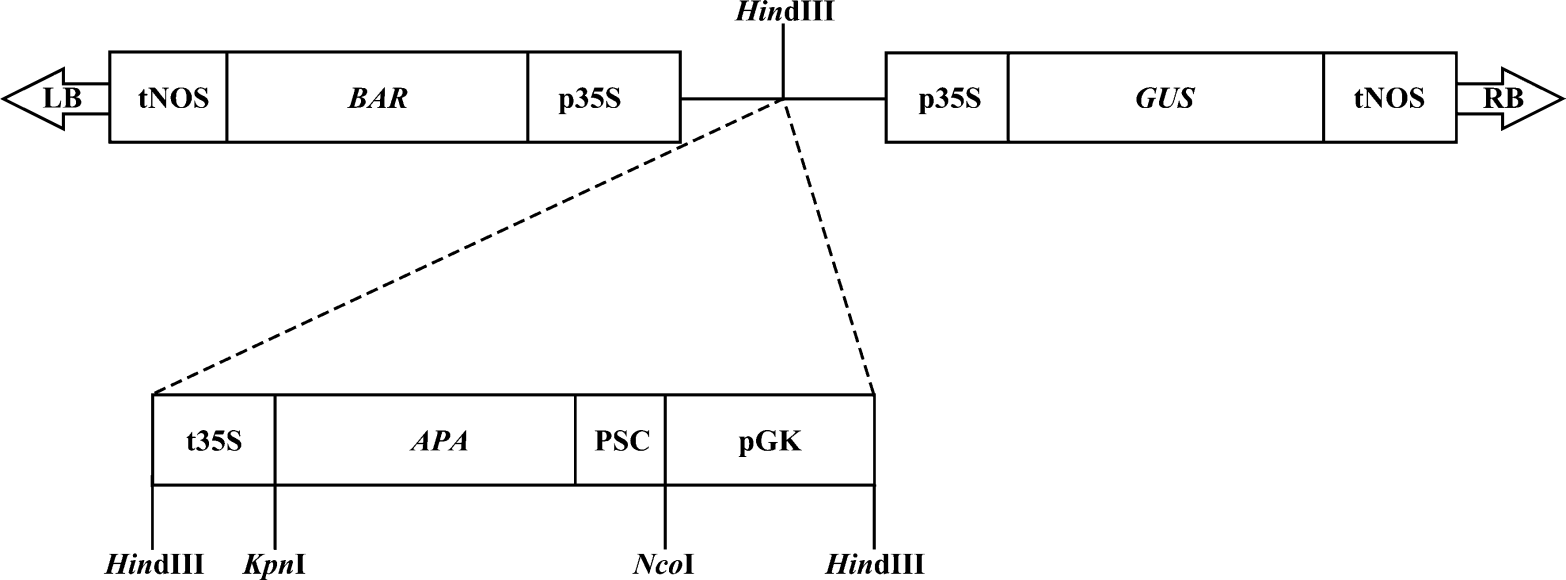
Structure of the pCam-PSC-APA vector used for tobacco genetic transformation. The *M. tuberculosis APA* gene (*APA*) was fused with α-coixin signal peptide (PSC) under the control of γ-kafirin promoter (pGK) and the 35S terminator (t35S). The *BAR* and *GUS* genes are under the control of the 35S promoter (p35S) and the terminator from the *NOS* gene (tNOS). LB and RB are the borders of the expression vector

The APA coding region (GenBank Accession Number X80268) was fused to the α-coixin signal peptide from *Coix lacryma*-*jobi* to target the recombinant protein to the endoplasmic reticulum (Ottoboni et al. 1993). The DNA encoding the α-coixin signal peptide (named PSC) was produced by annealing two synthetic oligonucleotides: PS1 (5′-CATGGCTACCAAGATATTTGCCCTCCTTGTGCTCCTTGCTCCTTGTGCTCCTTGCTCTTTCAGCGAGCGCTACAACTGCG-3′) and PS2 (5′-CGCAGTTGTAGCGCTCGCTGAAAGAGCAAGGAGCACAAGGAGGGCAAATATCTTGGTAGC-3′), giving rise to a fragment with 5′ *Nco*I overhang and a 3′ blunt end. The *APA* 871 bp fragment was amplified by PCR using the primers TBLEFT (5′-GATCCGGAGCCAGCGCC-3′) and TBRIGHT (5′-CGGGATCCCGTCAGGCCGGTAAGGTCCGCT-3′; *Bam*HI site underlined) using as template the pLA34 plasmid containing the *APA* sequence (Laqueyrerie et al. 1995). The PSC fragment was fused to the blunt end of the amplified *APA* fragment, resulting in the fragment named PSC-APA, containing a 5′ *Nco*I overhang, which was later digested with *Bam*HI. The PSC-APA fragment was inserted into the pRT-PGK (Leite et al. 2000) vector previously digested with *Nco*I/*Bam*HI, giving rise to the plasmid pRT-PGK-PSC-APA. Subsequently, a *Hin*dIII fragment of pRT-PGK-PSC-APA containing the expression cassette with the seed specific γ-kafirin promoter (PGK) from sorghum (de Freitas et al. 1994) and the 35S terminator was inserted into the *Hin*dIII site of pCambia 3301 vector (CAMBIA, Australia), giving rise to the plasmid pCam-PSC-APA, which additionally has, in the T-DNA, the *BAR* gene responsible to encode the phosphinothricin-N-acetyl transferase enzyme, that confers resistance to the herbicide phosphinothricin (PPT) to plants, and the reporter *GUS* gene, encoding β-glucluronidase, both under control of the constitutive promoter 35S (Fig. 1). The pCam-PSC-APA plasmid was transferred to *Agrobacterium tumefaciens* GV3101 (Koncz and Schell 1986) by thermal shock.

### Plant transformation

The transformed *Agrobacterium* was cultivated in 20 mL of LB broth with antibiotics (100 mg/L rifampicin, 60 mg/L gentamicin and 100 mg/L kanamycin) at 28 °C until reaching an OD_600_ of 0.8. After brief centrifugation, the cell pellet was resuspended in 1 mL of 0.85% NaCl solution at and kept at room temperature before use.

Leaves from tobacco plants grown as described above were cut in 0.5 to 1.0 cm^2^ fragments and then were cultured in 20 mL of INFTAB medium (MS salts, 100 mg/L myo inositol, 1 mg/L calcium pantothenate, 1 mg/L nicotinic acid, 1 mg/L pyridoxine, 1 mg/L thiamine, and 0.01 mg/L biotin) with 200 μL of *Agrobacterium* GV3101 containing the pCam-PSC-APA construct. After 48 h in the dark at 25 °C, leaf fragments were transferred to solid INFTAB medium containing 1 mg/L benzylaminopurine, 300 mg/L timentin and 5 mg/L phosphinothricin. Shoots were generated after 4–5 weeks, isolated and transferred to MS medium with 300 mg/L timentin and 5 mg/L phosphinothricin for root formation. Tobacco plants with roots were transferred to a greenhouse after 3–4 weeks for acclimatization. After verifying the genomic integration of the *APA* by PCR and the expression of the *GUS* transgene, the seeds were collected for further analysis.

### Detection of *APA *gene in transgenic plants

Genomic DNA was isolated from transformed and wild-type tobacco leaves by the CTAB method (Murray and Thompson 1980). The presence of the *APA* gene in the genomic DNA was confirmed by PCR using the forward primer TBLEFT and the reverse primer TBRIGHT.

### Detection of the recombinant APA protein from transgenic tobacco plants

For protein extraction, 50 mg of transgenic seeds were homogenized by grinding in a mortar with 1 mL of extraction buffer (50 mM Tris–HCl, pH 8.0, 2 mM EDTA, 5 mM benzamidine, 5 mM dithiothreitol, 0.5% Triton X-100). Homogenates were centrifuged at 10,000×*g* for 10 min at 4 °C. The supernatant was collected and mixed with hexane (1:1 v/v) and centrifuged at 15,000×*g* for 15 min at 4 °C. The lower phase was collected and protein concentration was determined using the BSA Protein Assay Kit (Pierce, USA).

An aliquot of 40 μg of the soluble proteins was mixed with sample buffer (125 mM Tris HCl, 4% SDS, 10% β-mercaptoethanol, 20% glycerol, 0.04% bromophenol blue, pH 6.8) and the samples were denatured in water at 95 °C for 5 min. The samples were separated by denaturing SDS-PAGE using a 12% polyacrylamide gel. Proteins were electrophoretically transferred to a nitrocellulose membrane (Hybond-C Extra, GE Healthcare, USA) using a Trans-blot SD semi-dry transfer cell (Bio-Rad, USA). Membranes were blocked with TBS (25 mM Tris–HCl, 150 mM NaCl, 2 mM KCl, pH 7.4) with 10% non-fat dry milk overnight at 4 °C. Then, membranes were incubated with gentle agitation for 2 h in 20 mL of TBS with 5% non-fat dry milk with an anti-APA rabbit polyclonal antibody, kindly provided by Dr. G. Marchal (Pasteur Institute, France) (Laqueyrerie et al. 1995), diluted 1:4000. Membranes were washed three times with TBS-T (TBS, 0.005% Tween 20) and three times with TBS, followed by an incubation with a 1:20,000 dilution of anti-rabbit IgG conjugated with peroxidase (Pierce, USA) in 20 mL TBS with 5% of non-fat dry milk for 2 h with gentle agitation. Then, membranes were washed three times with TBS-T and TBS. The chemiluminescent signal was developed with Super Signal^®^West Pico (Pierce, USA) and the signal was revealed using X-ray films. The native APA protein from *M. tuberculosis,* kindly provided by C. Horn (FIOCRUZ, Brazil) (Horn et al. 1999) was used as positive control, and protein from non-transformed tobacco seeds were used as negative control.

### Concanavalin binding assay

The affinity of the recombinant APA protein for concanavalin was determined by affinity chromatography. ConA Sepharose (GE Healthcare, DE) resin was washed in equilibrium buffer (20 mM Tris–HCl, 0.5 M NaCl, pH 7.4) and 5 mL of resin were incubated with 100 mL of soluble proteins overnight at 4 °C with gentle agitation in a blood homogenizer. The mixture was transferred to an Econo-Column chromatography column of 1.5 × 10 cm (Bio-Rad, USA) and washed with 50 mL of equilibrium buffer, using a flow rate of 1 mL/min to elute non-bound protein. Bound proteins were eluted with 0.01 M borate buffer at pH 7.4 and 0.1 M borate buffer at pH 7.4 under continuous flow (30 mL/h). Eluted protein fractions were collected and stored at − 20 °C until used for silver-staining SDS-PAGE and 2D electrophoresis western blot.

### Two-dimensional electrophoresis

For two-dimensional electrophoresis, 25 μg of concanavalin-bound proteins obtained above were solubilized in 125 μL of rehydration buffer containing 8 M urea, 2% CHAPS, 2% tributylphosphine (TBP), 1% carrier ampholytes, 0.01% bromophenol blue (w/v). Samples were incubated for 1 h at room temperature and loaded onto an immobilized pH gradient (IPG) strip, pH 3–10. Isoelectric focusing was performed on a Protean^®^ IEF cell (Bio-Rad, USA) with maximum current of 50 μA/strip. Focusing parameters used for IPG strips in the pH range 3–10 were: active rehydration (50 V) for 11 h; step 1—linear gradient from 1 to 250 V over 20 min; step 2—linear gradient from 250 to 4000 V over 2 h; step 3—constant 4000 V until 18,500 Vh was achieved. The strips were then incubated for 15 min in equilibration buffer (50 mM Tris–HCl, 6 M urea, 30% glycerol, 2% SDS, pH 8.8) containing 25 mM dithiothreitol with gentle agitation, followed by incubation for 15 min in equilibration buffer containing 55 mM iodoacetamide. Then, the strips were transferred to a 12% SDS polyacrylamide gel (1.5 mm thick) and the focalized proteins were separated by electrophoresis, in standard Laemmli buffer at 100 mA/gel, until the tracking dye left the gel. The recombinant protein was detected by western blot as described previously.

## Results

### Production of transgenic plants containing the *APA* gene

The *APA* gene from *M. tuberculosis* was amplified by PCR and linked to a synthetic α-coixin signal peptide DNA sequence to enable the targeting of the recombinant protein to the endoplasmic reticulum and accumulation in protein storage vacuoles. This fusion, named PSC-APA, was inserted into the PRT-PGK plasmid under the control of the γ-kafirin seed-specific promoter and the 35S terminator. The expression cassette was cut from this plasmid and inserted into the plant expression vector pCambia 3301. This vector, named pCam-PSC-APA (Fig. 1), was introduced into *Agrobacterium tumefaciens* GV3101 to produce transgenic plants. We selected, for further analysis, seven independent plants with no visible morphological changes that grew under a selection media containing phosphinothricin, indicating the tobacco genetic transformation by the expression of the *BAR* gene present in T-DNA.

**Fig. 2:**
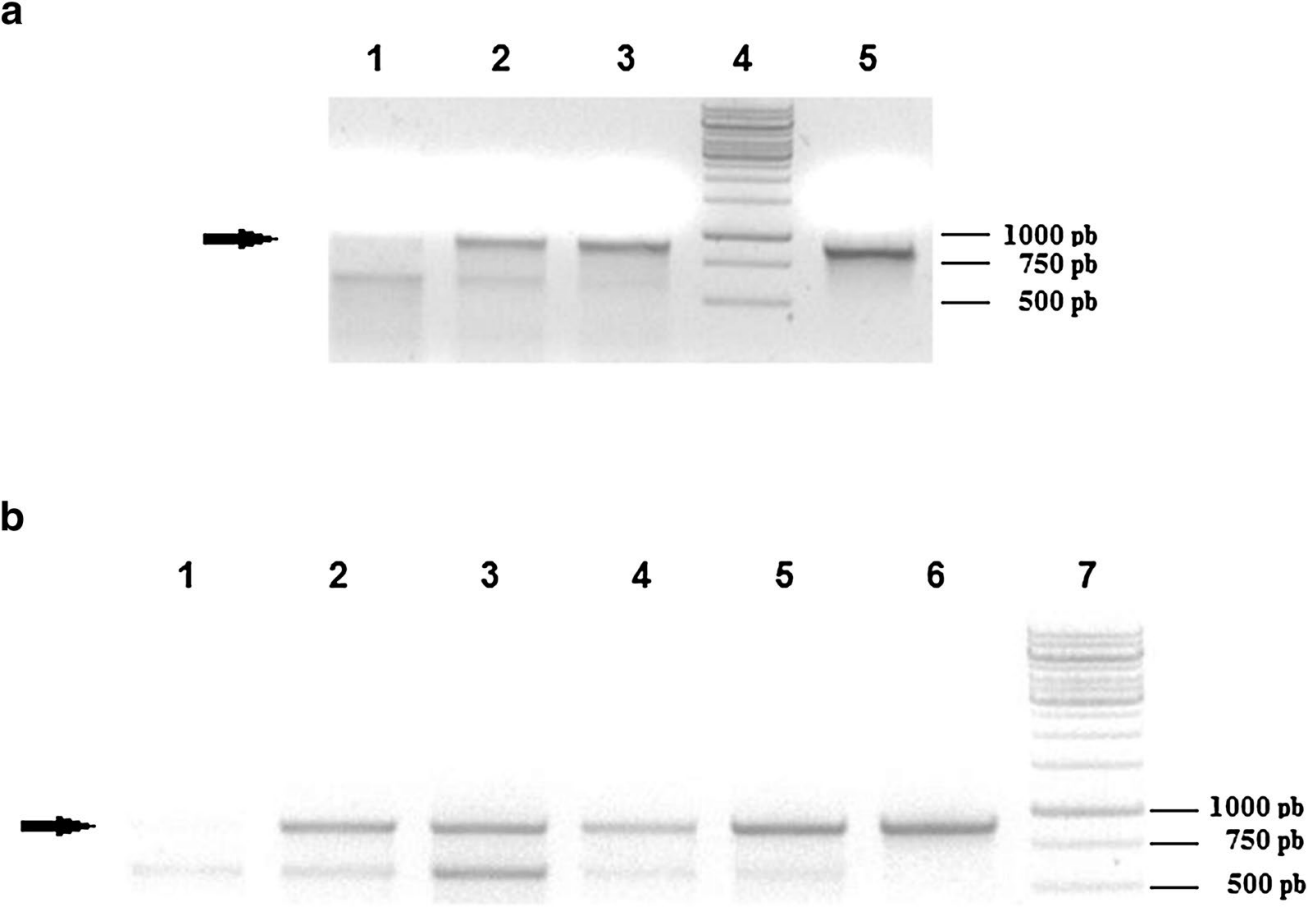
Detection of the *APA* gene in transgenic tobacco plants. Genomic DNA was amplified with specific primers to detect an 871 bp fragment (indicated by an arrow) corresponding to the *APA* gene. **a** Lane 1: negative control; Lanes 2 and 3: transgenic plants; Lane 4: DNA size marker; Lane 5: positive control. **b** Lane 1: negative control; Lanes 2–5: transgenic plants; Lane 6: positive control; Lane 7: DNA size marker. The negative control was the amplification of the genomic DNA from wild-type plants; the positive control was the amplification from pCam-PSC-APA plasmid DNA and the DNA size marker used was the Gene Ruler 1 kB (Fermentas, USA)

The presence of the APA coding sequence in these plants was confirmed by PCR of genomic DNA using *APA* gene specific primers (Fig. 2). The positive transgenic plants were identified by the presence of an 871 bp DNA fragment. The transgenic plants showed the same size fragment observed in the sample containing the pCam-PSC-APA vector used as positive control. A non-specific PCR product of approximately 650 bp was observed in all plants, including the wild-type, non-transformed plants (Fig. 2). All transgenic APA plants were also positive for *GUS* gene expression, present in the T-DNA, while wild-type plants showed no GUS activity (data not show).

### Detection of recombinant APA protein in transgenic tobacco plants

**Fig. 3:**
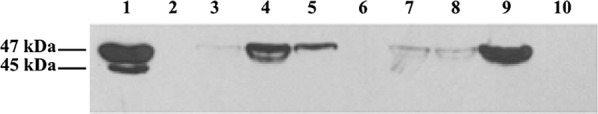
Detection of the APA recombinant protein in tobacco plants. An anti-APA polyclonal antibody was used to detect the APA protein. Lane 1: 500 ng of APA purified from *M. tuberculosis* as positive control. Lane 2: Empty lane; Lanes 3–9: protein extracts from transgenic seeds. Lane 10: protein extract from wild-type *Nicotiana tabacum* seeds (negative control). The positions of the expected bands of 47 and 45 kDa are indicated on the left

Total soluble proteins from seeds of the seven transgenic plants selected by PCR and GUS activity were tested to detect the presence of the recombinant APA protein by western blot, using a rabbit anti-APA polyclonal antibody. As shown in Fig. 3, the native APA protein from *M. tuberculosis,* used as positive control, showed the characteristic 45 and 47 kDa bands, as described previously (Romain et al. 1993). The presence of a 47 kDa band was observed in the transgenic plant samples, indicating that the recombinant APA protein was similar to the APA protein from *M. tuberculosis* (Fig. 3). Additionally, it is possible to visualize a band below 47 kDa with approximately 46 kDa (Fig. 3, lanes 4, 7 and 8) when lower amounts of recombinant protein was detected, while in the plant with higher recombinant protein production the two bands appear to overlap due to the strong signal of the detected protein (Fig. 3, lane 9). The events with the highest expression were T-APA1 and 4 (Fig. 3, lanes 9 and 4 respectively). No band corresponding to the APA was detected in the protein extract from the wild-type *N. tabacum*, indicating the high specificity of the anti-APA antibody utilized in this work (Fig. 3, lane 10).

### Concanavalin binding assay

**Fig. 4:**
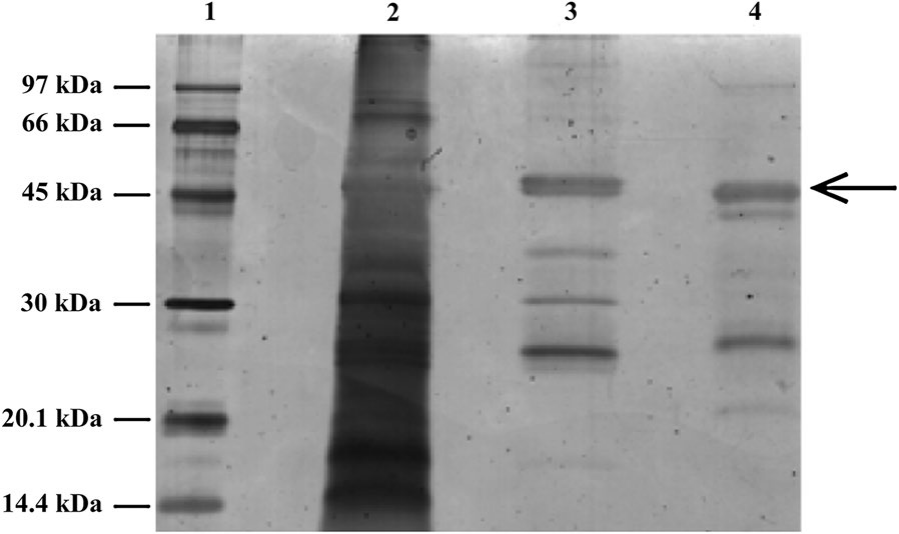
Silver-staining SDS-PAGE from tobacco proteins purified with concanavalin affinity chromatography. Lane 1: Low molecular weight marker (GE Healthcare, UK); lane 2: total soluble proteins from transgenic tobacco seeds (event T-APA1); lane 3: eluted fraction after ConA Sepharose purification; lane 4: 500 ng of purified native APA protein from *M. tuberculosis*. The position of the recombinant APA protein is indicated with an arrow

Recombinant APA from transgenic tobacco seeds was semi-purified by affinity chromatography using ConA Sepharose, a resin containing concanavalin that binds glycoproteins. Protein extracts from transgenic tobacco seeds were applied to the resin, and aliquots of 1 mL were collected. The presence of the recombinant APA protein and contaminating proteins in these fractions was determined by silver-staining SDS-PAGE (Fig. 4), due the high sensitivity of this technique to stain proteins even in low amounts. We observed two bands with approximate sizes of 46 and 47 kDa, corresponding to recombinant APA, and other bands with approximately 25, 30, 37 kDa which are probably contaminating proteins from tobacco seeds that were also co-eluted with APA (Fig. 4, lane 3). It is also shown that the APA control protein has some contaminant proteins, probably due to incomplete purification of the native protein from *M. tuberculosis.* Although the APA protein eluted in ConA affinity chromatography presented a low amount of contaminating proteins when compared to the protein extract of transgenic tobacco seeds, future steps of purification polishing can be applied in order to obtain a recombinant protein with a higher degree of purity.

### Two-dimensional electrophoresis

**Fig. 5:**
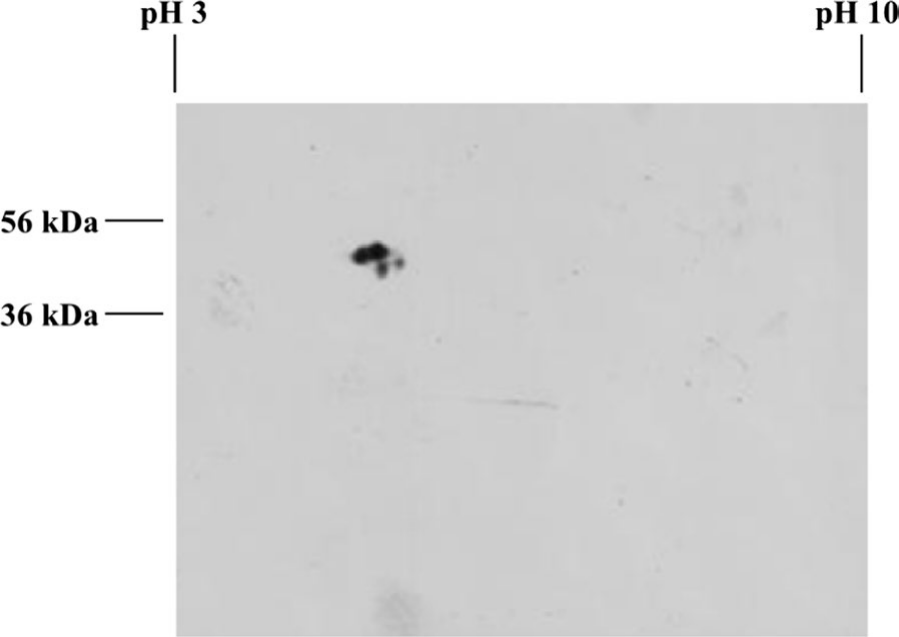
Western blot of two-dimensional electrophoresis of affinity chromatography purified recombinant APA protein. The electrofocusing was performed on a linear IPG (pH 3.0 to 10.0). The pH gradient is indicated on the top of the figure and the molecular size marker on the left

To check the heterogeneity of the recombinant APA protein and its isoforms, the semi-purified protein fraction shown in Fig. 4, after ConA Sepharose chromatography, was analyzed by 2D electrophoresis. The identification of the recombinant protein was performed by western blot using a polyclonal antibody against the *M. tuberculosis* APA protein (Fig. 5). Four spots were observed, indicating the presence of two isoforms in the 47 kDa and 46 kDa recombinant proteins, with distinct isoelectric points between 4.0 and 5.0 (Fig. 5). This result confirmed the presence of the 47 and 46 kDa protein complex in the SDS-PAGE western blot (Fig. 3) and in the fraction purified by affinity chromatography, indicating that the recombinant APA protein has more than one isoform, probably because of changes in the glycosylation pattern.

## Discussion

The low efficacy of BCG vaccine in protecting against pulmonary TB in adults and the risk of immunocompromised individuals developing the disease by BCG vaccination are major drivers to the development of an effective subunit vaccine against *M. tuberculosis*. One way to obtain these antigens is the expression of recombinant proteins in transgenic organisms. Plants are an interesting alternative due to their advantages compared to other systems used for the production of recombinant proteins, because of their lower costs for cultivation when compared with expensive growth media used to mammalian and insect cells cultivation, the absence of contamination of the product by mammalian pathogens and bacterial endotoxins and their scalability (Ma et al. 2003; Schillberg et al. 2003).

Previous studies with a plantibody expressed in tobacco seeds found that this species is able to glycosylate recombinant proteins (Hernández-Velázquez et al. 2015). The plantibody contained structures of high-mannose and predominantly type-complex N-glycans, indicating that a high proportion of molecules modified with α (1.3) fucose and β (1.2) xylose. Additionally, a high mannose type N-glycan profile of five to nine mannoses residues was found, and the most prevalent modification was the presence of seven mannose residues in the recombinant protein, with a oligomannoside structure proportion of 52% of the plantibody N-glycans (Hernández-Velázquez et al. 2015).

In this study we produced transgenic tobacco plants that were able to accumulate recombinant APA protein. Recombinant APA proteins with 46/47 kDa were detected by SDS-PAGE western blot, and four spots were observed when separated in two-dimensional electrophoresis immunobloting, similarly as described previously in protein extracts from *M. tuberculosis* (González-Zamorano et al. 2009) and *M. bovis* BCG (Romain et al. 1993; Ragas et al. 2007).

The antigen APA is described as a Concanavalin A (ConA) binding protein due its affinity with ConA (Espitia et al. 1995; Lara et al. 2004; González-Zamorano et al. 2009), a lectin carbohydrate linker. We found evidences of glycosylation of the recombinant APA expressed in tobacco seeds due to the ability to bind ConA by affinity chromatography, demonstrating that the recombinant protein was glycosylated. Other glycosylated proteins found in plant seeds also have ConA affinity and were eluted together with the recombinant protein (Fig. 4) and were not detected by two-dimensional western blot using a specific anti-APA antibody (Fig. 5). Although we observed the binding of other proteins in the chromatography, the use of ConA affinity was effective for the partial purification of the APA antigen.

Previous studies describe the 45 kDa protein being a product derivative from a proteolytic action on the of 47 kDa protein, which lacks a C-terminal glycosylated peptide (Romain et al. 1999). However, this statement has never been demonstrated definitely. Interestingly, the reduced number of glycans in the 45 kDa protein drastically reduces its affinity for PSP-A (human pulmonary surfactant protein A), a C-type lectin (Ragas et al. 2007).

Interestingly, when the APA protein is expressed in *S. lividans*, the 45/47 kDa proteins were observed in SDS-PAGE (Lara et al. 2004), but the 45 kDa form did not react with ConA, indicating that this recombinant protein form is glycosylated by sugars other than mannose or by a different configuration that is not recognized by ConA. Therefore, these authors suggested that even for the native 45/47 kDa protein, the existence of two bands can be attributed due to differences in glycosylation of the same protein instead of a proteolytic action or C-terminal modification (Lara et al. 2004). In contrast to the *S. lividans* recombinant APA, the plant-made 46/47 kDa protein had affinity for ConA, and the two bands were eluted together after the affinity chromatography, indicating that both forms were glycosylated with ConA binding glycans.

Regarding the 45/47 kDa proteins expressed in *S. lividans*, these authors observed that the recombinant protein had the same isoelectric point, which differs from the native proteins pattern that migrate as several spots in 2D gel electrophoresis due to differences in their isoelectric points, caused by a diversity in glycosylation on native APA (Espitia et al. 1995; Lara et al. 2004). According to the results of our bi-dimensional gel electrophoresis followed by western blot, we estimated that the pI of the APA protein obtained from transgenic plants ranged from 4 to 5. The presence of four spots from the 46/47 kDa complexes suggests the presence of four isoforms of the protein. The presence of different APA isoforms in the native APA preparations has been extensively described for both 45 and 47 kDa protein in bi-dimensional gel (Romain et al. 1993; Espitia et al. 1995; Ragas et al. 2007).

Taking into account that differences in protein glycosylation may lead to a different protein migration in both SDS-PAGE and bi-dimensional electrophoresis, we conclude that the presence of two different mass in SDS-PAGE and four spots in 2D western blot is due to a diverse glycosylation pattern of the recombinant plant APA. This pattern can also reflect a distinct number of mannose residues, as observed for the N-glycosylation in tobacco seeds by Hernández-Velázquez et al. (2015). It is worth noting that the APA protein has two N-glycosylation sites (Asn-X-Ser/Thr) in its primary structure, which allows the presence of this type of glycosylation in this protein, in addition to the regions prone to O-glycosilation.

Many reports on plant-made vaccines for human and animal health (reviewed by Laere et al. 2016; Rybicki 2017) show the increased interest in the development of new strategies for the use of plants as bioreactor for antigen production. In spite of the differences in protein glycosylation between plants and others heterologous protein expression systems, a large number of plant-made antigens has been proposed in the literature. We have produced transgenic tobacco plants expressing a glycosylated APA protein that resembles the native protein. We emphasize that future characterization of the glycan structure of the plant made APA will be helpful to compare the structure and biological properties of native bacterial products to those obtained by recombinant DNA technology. Our data open new avenues for the production of a recombinant protein to be used for prevention against tuberculosis.

## Abbreviations

APA: Alanine- and Proline-rich antigen
BCG: Bacillus Calmette–Guérin
Con A: Concanavalin A
ESAT-6: early secretory antigenic target
FAP: fibronectin attachment protein
MS: Murashige and Skoog medium
SDS-PAGE: Sodium dodecyl sulfate–polyacrylamide gel electrophoresis
TB: tuberculosis
